# Supplementary material for: A rotary plasmonic nanoclock
Source: Nat Commun. 2019 Nov 27;10:5394. doi: 10.1038/s41467-019-13444-3 (PMC6881389; doi:10.1038/s41467-019-13444-3)
Supplement: Supplementary file 1 — Supplementary Information [file 41467_2019_13444_MOESM1_ESM.pdf]

Supplementary Information for

A rotary plasmonic nanoclock

Xin et al.

Correspondence to: na.liu@kip.uni-heidelberg.de

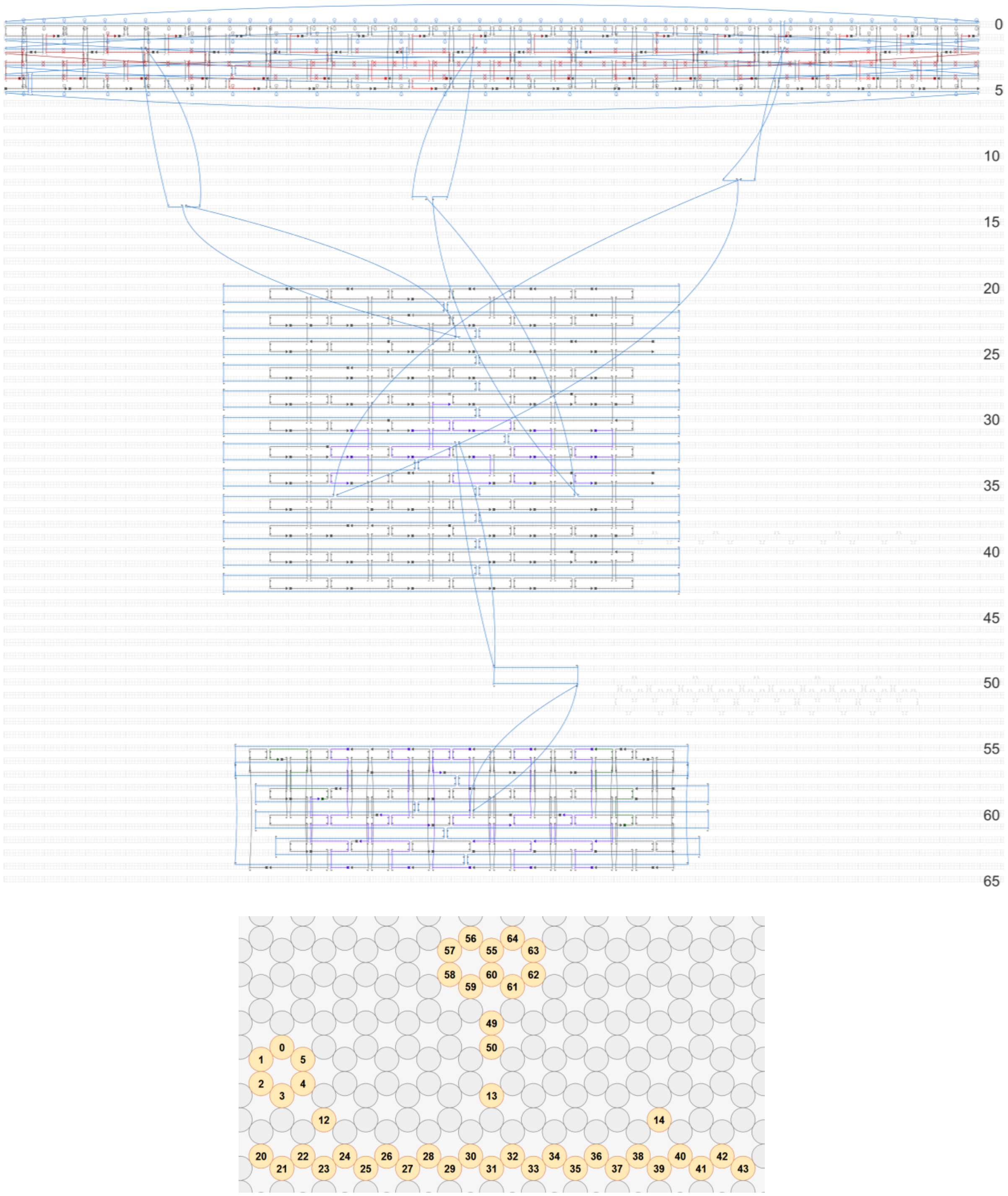

Supplementary Figure 1| Strand routing diagram of the DNA origami structure for the stepwise plasmonic nanoclock. Staples for two foot strands (green), 16 footholds (red), 20 capture strands (violet).

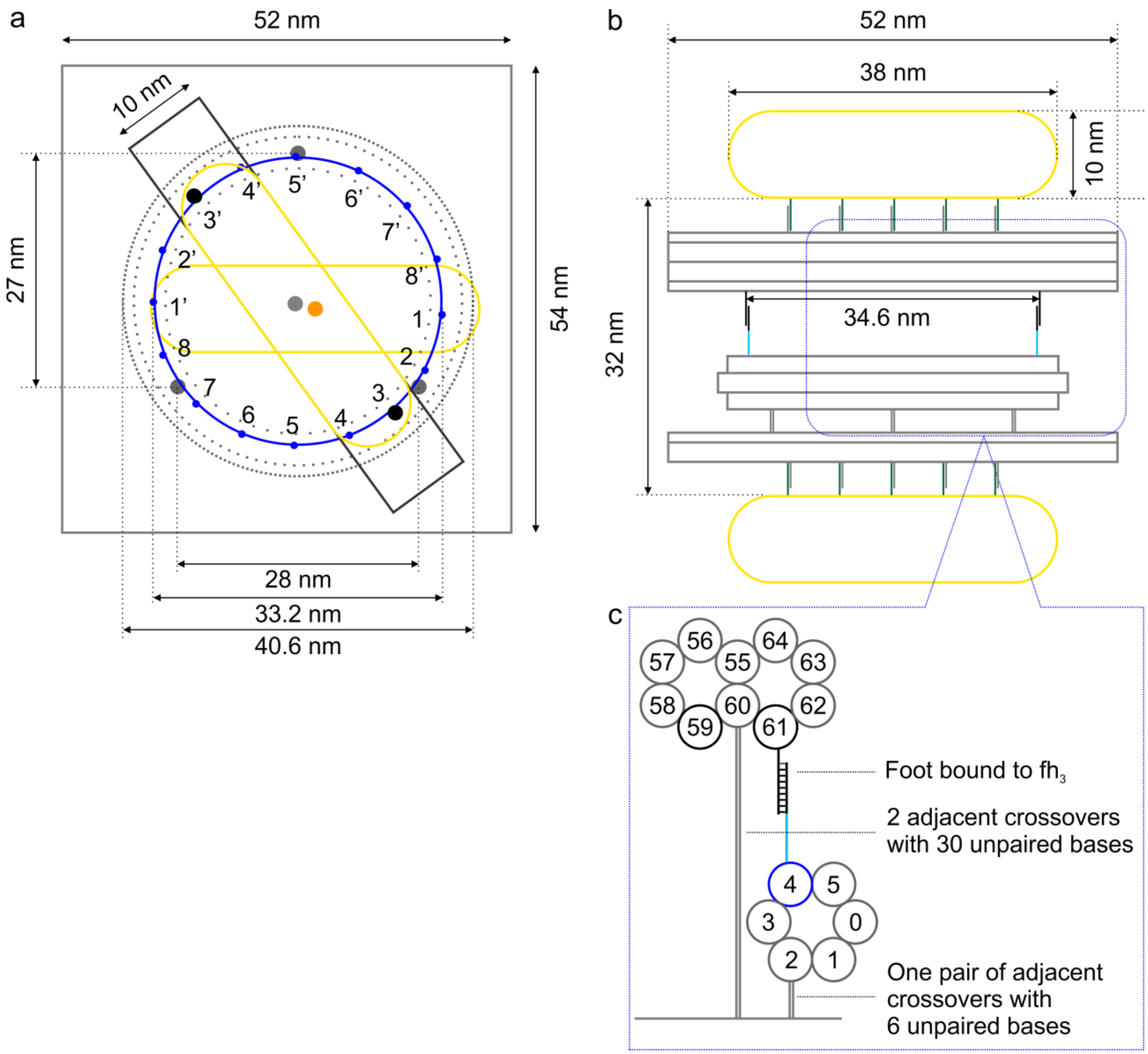

Supplementary Figure 2 | Structural details of the stepwise plasmonic nanoclock. **a**, Top and **b**, Side views of the plasmonic nanoclock at position 3-3′. Three grey dots under the blue ring in the top view represent the positions of the three pairs of adjacent crossovers with 6 unpaired bases to connect the lower surface of the 6-helix ring and the upper surface of the single-layer plate. The grey dot in the center of the circle represents the position of 2 adjacent scaffold crossovers with 30 unpaired bases used to connect the centers of the 10-helix bundle and the single-layer plate, which are not shown in the side view. The orange dot is the center of the capturing strands extended from the bottom of the plate for attaching the stator AuNR. The orange dot and grey dot don′t coincide with each other (the lateral distance between them is ~ 2.3 nm), meaning the two AuNRs are not coaxial. The four circles in the top view represent the positions of the different helices in the ring. The crossovers used to immobilize the ring on the plate are distributed on helix 2 and the footholds are extended from helix 4. Both helices are along the blue circle in the top view. The origami bundle is about 10 nm in width and 52 nm in length. The two black dots along the diagonal direction of the rotor represent the two feet and coincide with the two small blue dots at position 3-3′, meaning the two feet are bound to fh_3 and $fh_{3'}$, respectively. The rotor is fixed at position 3-3′. **c**, Enlarged view of connections between ring track, rotor and plate. Scaffold crossovers are used to immobilize the rotor and ring on the plate. Meanwhile, the rotor and ring track are linked via foot-foothold interaction.

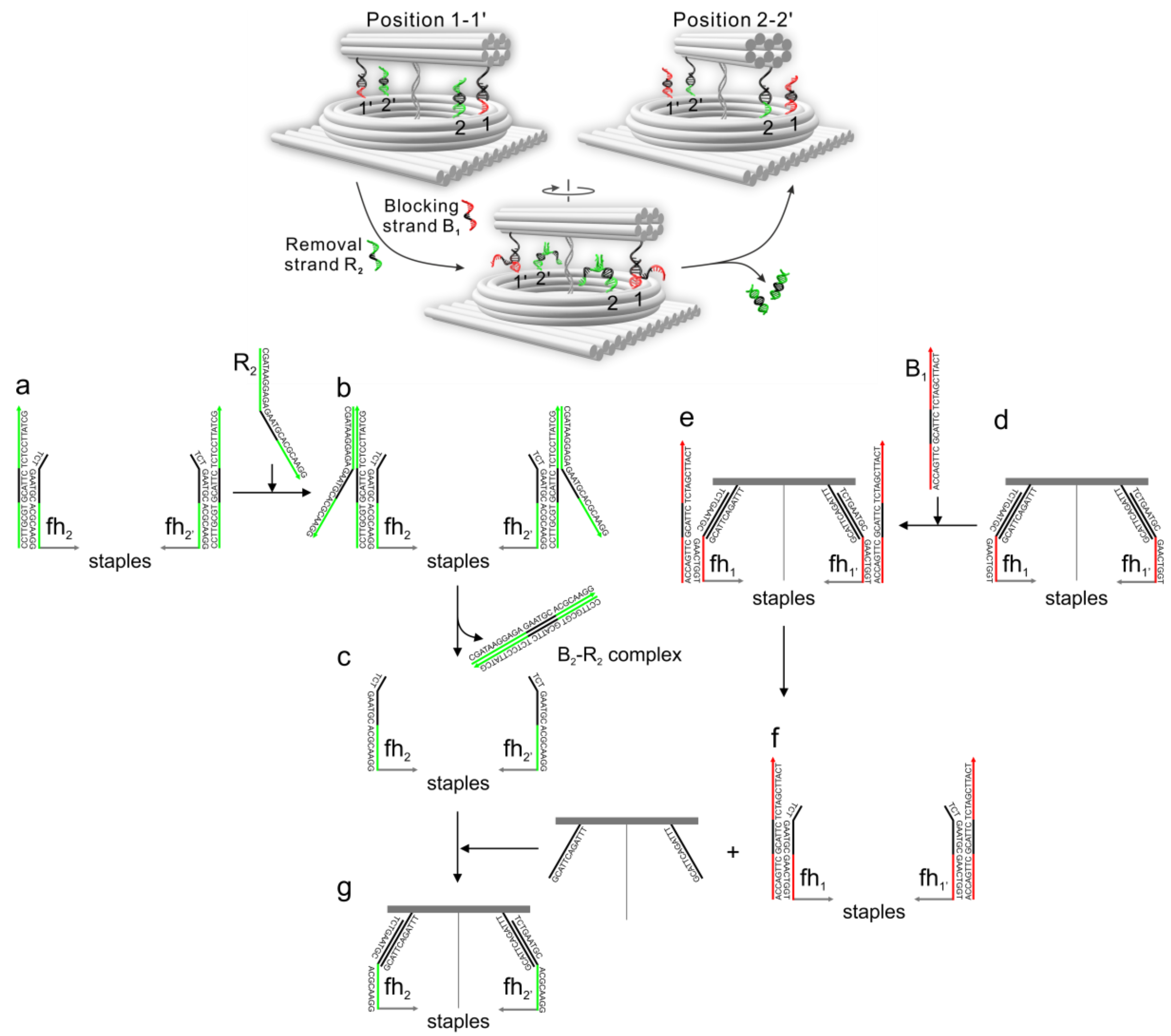

Supplementary Figure 3 | Schematic illustration of the stepwise rotation from position 1-1′ to position 2-2′. Each pair of the colored foothold strands on the ring track has a specific sequence. The two feet at the ends of the DNA bundle interact with a given pair of the footholds to determine the rotor position. For example, at the initial state the rotor is fixed at position 1-1′. At this state, the feet are bound to the red foothold pair (fh$_1$ and fh$_{1'}$) and all other footholds are blocked. (**a-c**) R$_2$ is first added to activate position 2-2′. B$_2$ is dissociated by R$_2$ through branch migration, triggered by top domain of B$_2$, and the green pair of the footholds (fh$_2$ and fh$_{2'}$) is activated. (**d-f**) Next, B$_1$ is added to block position 1-1′. Fh$_1$ and fh$_{1'}$ are deactivated by B$_1$ mediated through toehold domains on fh$_1$ and fh$_{1'}$. (**g**) Two feet are free for capturing fh$_2$ and fh$_{2'}$ and driving the rotor to position 2-2′. As a result, one step of the clockwise rotation is accomplished.

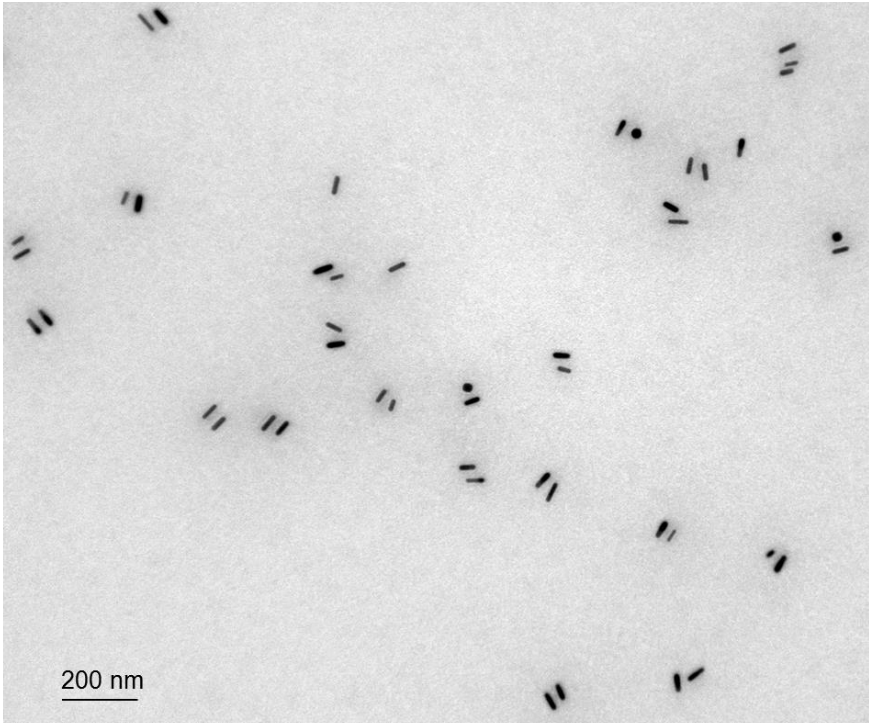

**Supplementary Figure 4 | **TEM image of the stepwise plasmonic nanoclocks at position 1-1′.

Theoretical calculations were performed using commercial software COMSOL Multiphysics based on a finite element method. The origin of the bisignate CD is a consequence of Coulomb interaction between the dipoles of the two AuNRs. The CD signal was calculated as a difference in extinction for the left- and right-circularly polarized light. Since the plasmonic assemblies were dispersed in a solution, we carried out orientational averaging. Averaging over all possible orientations at defined light incidence is equivalent to averaging over all incident directions of light for a nanostructure with defined orientation. It has been demonstrated both analytically and numerically that averaging over six orthogonal directions of light incidence is sufficient to give accurate CD. In order to account for the inhomogeneous broadening due to the polydispersity of the AuNRs, the experimental dielectric function of Au was modified by including an additional term:

$$\varepsilon_{\text{effective}}(\omega)=\varepsilon_{\text{bulk}}(\omega)+\varepsilon_{\text{correction}}(\omega) \quad (1)$$

where the dielectric function of bulk Au, $\varepsilon_{\text{bulk}}$ is from the Johnson and Christy values, and the correction term is introduced following a standard approach:

$$\varepsilon_{\text{correction}}(\omega)=\frac{\omega_{\text{p}}^2}{\omega^2+i\omega\gamma}-\frac{\omega_{\text{p}}^2}{\omega^2+i\omega\Gamma_{\text{broad}}} \quad (2)$$

ω_{p} = 8.754 eV and γ = 0.0724 eV are the Drude parameters, respectively. Γ_{broad} is 0.362 eV.

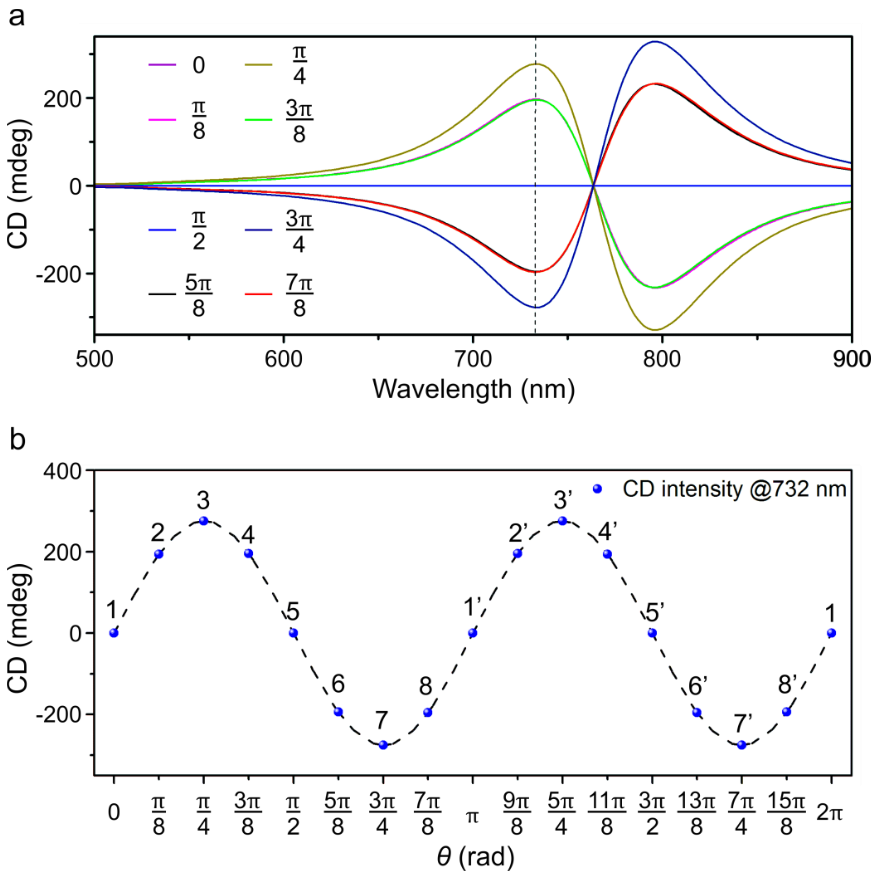

Supplementary Figure 5 | Theoretical results for the 16 states of the plasmonic nanoclock. **a**, Calculated CD spectra at different rotation angles. **b**, CD intensity at 732 nm as a function of the rotation angle *θ*.

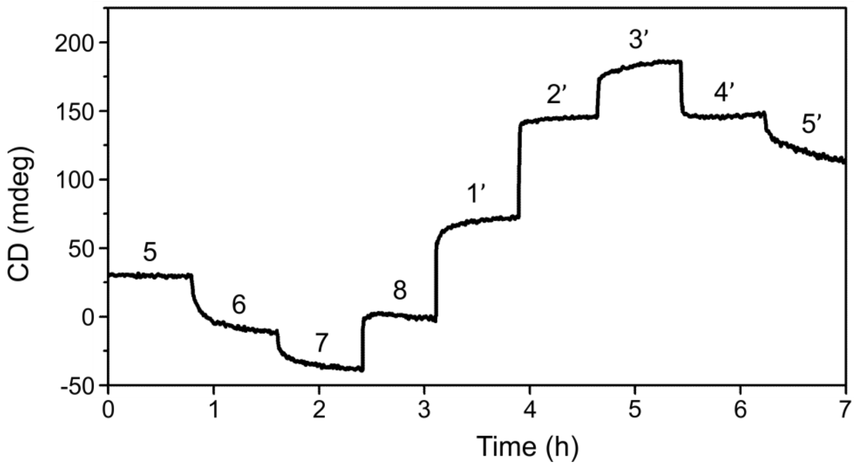

Supplementary Figure 6 | Kinetics of the stepwise rotations driven by corresponding DNA fuels. The concentration of the AuNRs is about 1.2 nM. This result also indicates that the plasmonic nanoclock can perform stepwise rotations from any starting position.

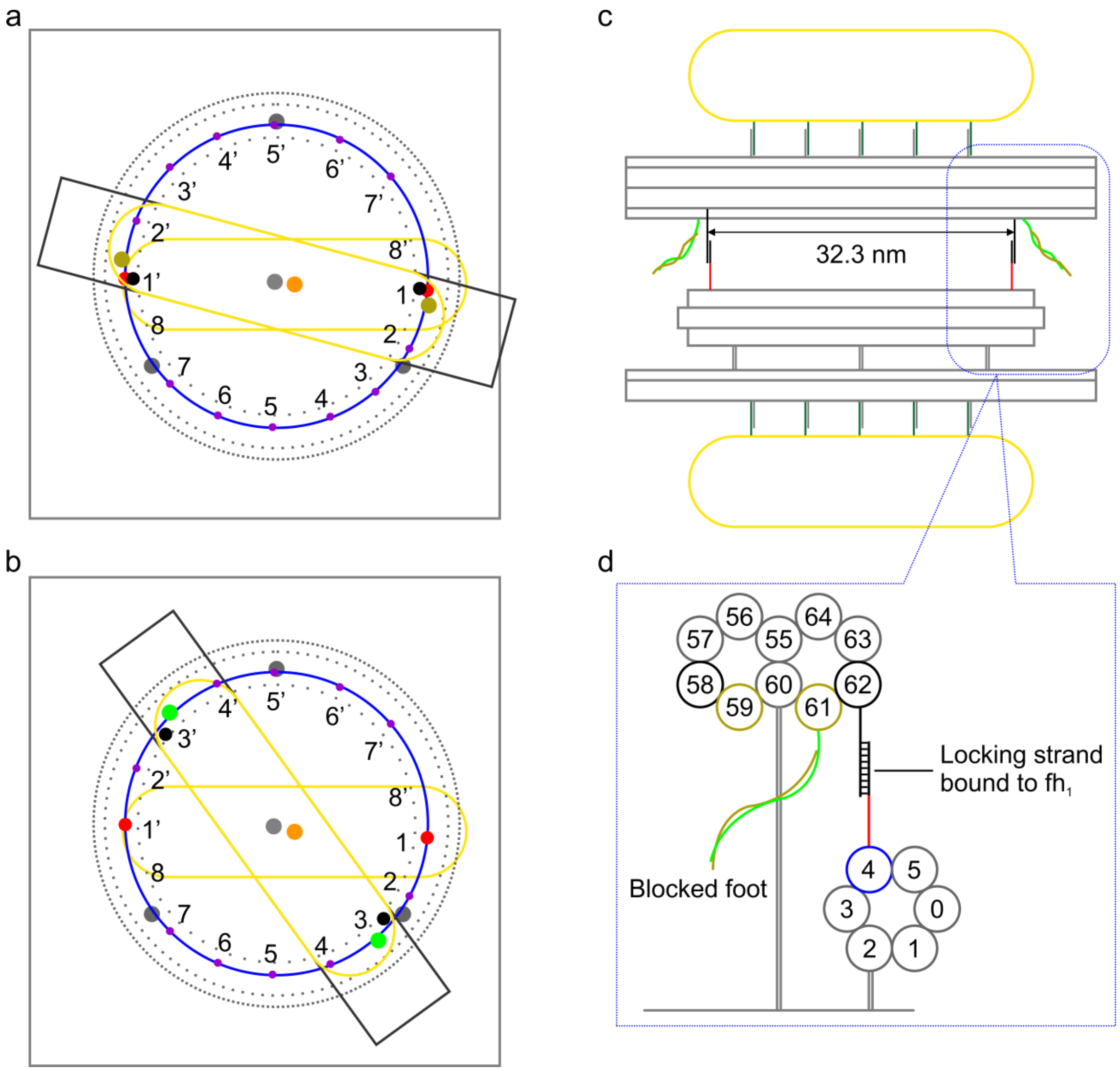

Supplementary Figure 7 | Structural details of the autonomous plasmonic nanoclock. Top-view of the plasmonic nanoclock with track arrangement of 1–2–3–4–5–6–7 at **a**, position 1-1′ and **b**, position 3-3′. Substrates (purple dots) as footholds are arranged on helix 4 (blue circle). Fh_1 and $fh_{1'}$ are working as lower locking strands (red dots). **a**, Two brown dots along the diagonal direction of the rotor represent that the DNAzyme feet are blocked. Two black dots next to the blocked feet represent the upper locking strands. They coincide with the red dots, which means the rotor is fixed at position 1-1′ through hybridization between the upper and lower locking strands. **b**, Two green dots represent the positions of the two activated feet superimposed with the two small purple dots at position 3-3′. The feet are bound to the substrates at fh_3 and $fh_{3'}$, and the rotor is fixed at position 3-3′. **c**, Side-view of the plasmonic nanoclock with track arrangement of 1–2–3–4–5–6–7 at position 1-1′ and the feet are blocked. The distance between the two upper locking strands is about 32.3 nm. **d**, Enlarged view of connection between ring track and rotor by the locking strands on helix 62 and 4. The foot strand (green) on helix 61 is blocked by blocking strand b (brown).

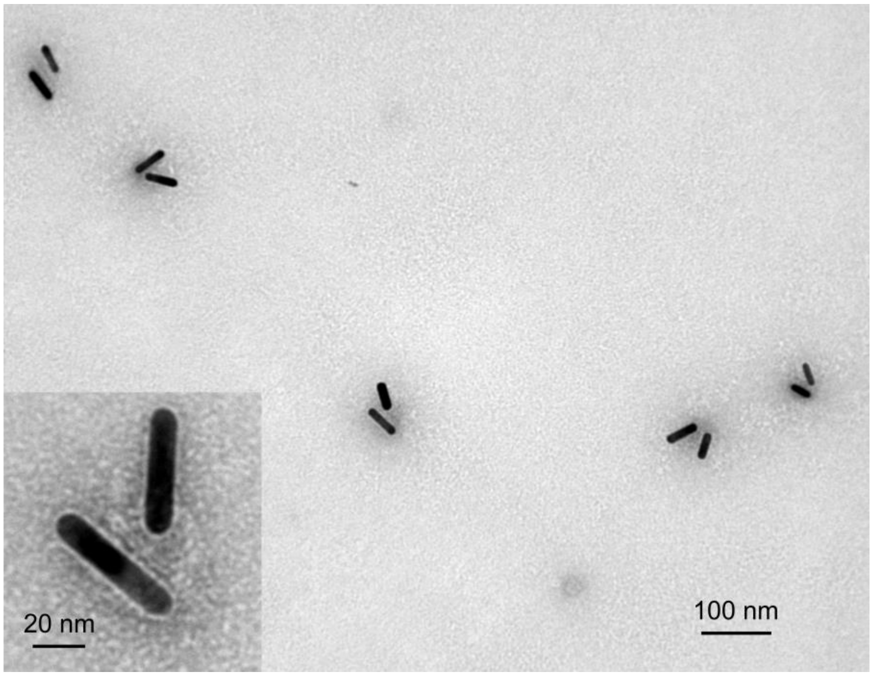

Supplementary Figure 8 │ TEM image of the autonomous plasmonic nanoclocks at position 1-1′.

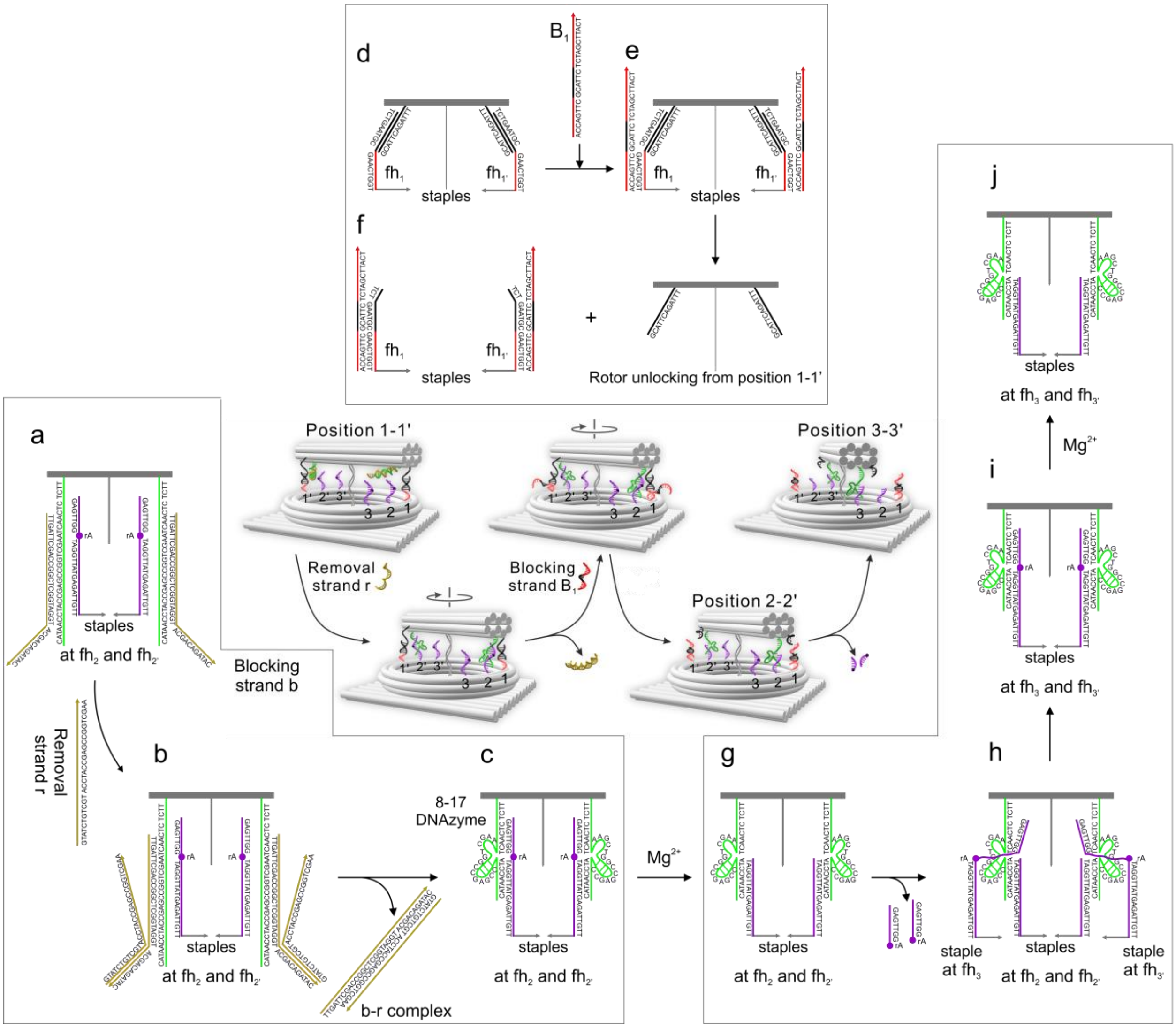

Supplementary Figure 9 | Schematic illustration of the autonomous rotation from position 1-1′ to position 3-3′. Before rotation, feet (green) are blocked by blocking strand b (brown). Each substrate (purple strand) contains a ribonucleotide (purple dot) in sequence at the 8 base from 5′ end. Two upper locking strands (black) next to feet hybridize with fh_1 and $fh_{1'}$ to fix the rotor at position 1-1′. The autonomous rotation is actuated through two sets of toehold-mediated strand displacement reactions. (**a-c**) Addition of removal strand r (brown), the feet are activated to interact with substrates at fh_2 and $fh_{2'}$. (**d-f**) B_1 is added to break the interactions between the locking strands and free the rotor for autonomous rotation. (**g-j**) In the presence of Mg^{2+}, the 8-17 DNAzyme in the foot strands catalyze the hydrolysis of the substrate at the RNA base (rA). The substrates are cut into two short segments (8-nt free segment and 17-nt staple extension). As the free segments diffuse in the solution, the adjacent pair of the intact substrates at fh_3 and $fh_{3'}$ are able to interact with the foot strands. Subsequently, the feet will move forward to position 3-3′. The processive rotation is therefore based on the burnt-bridge mechanism.

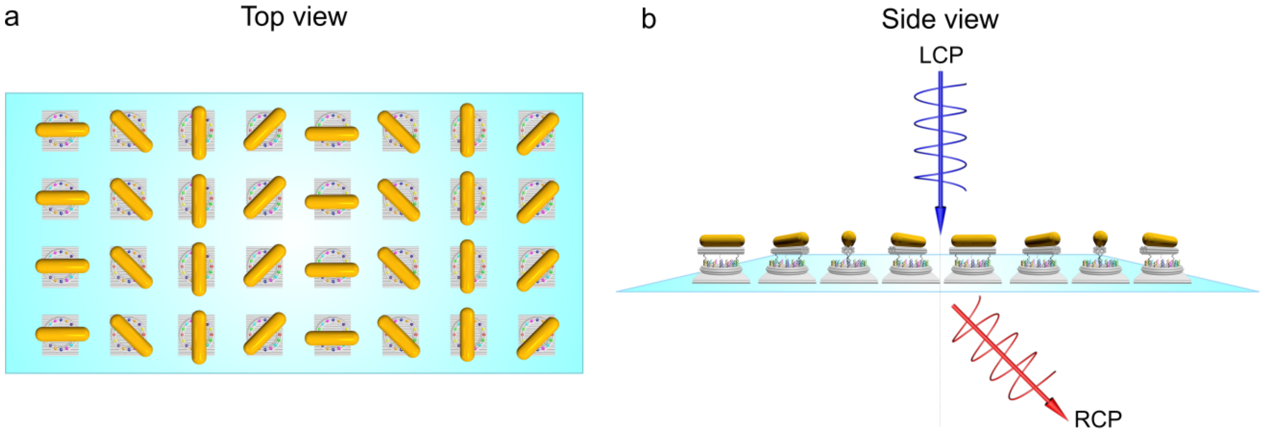

Supplementary Figure 10 | Visionary scheme of a dynamic optical metasurface, on which the DNA-assembled rotary AuNRs work as individual plasmonic pixels to dynamically shape the wavefront of light. The orientations of these AuNRs could be independently controlled by DNA nanotechnology. For instance, with a linear phase gradient distribution (**a**), anomalous refraction by the metasurface (**b**) could be achieved.

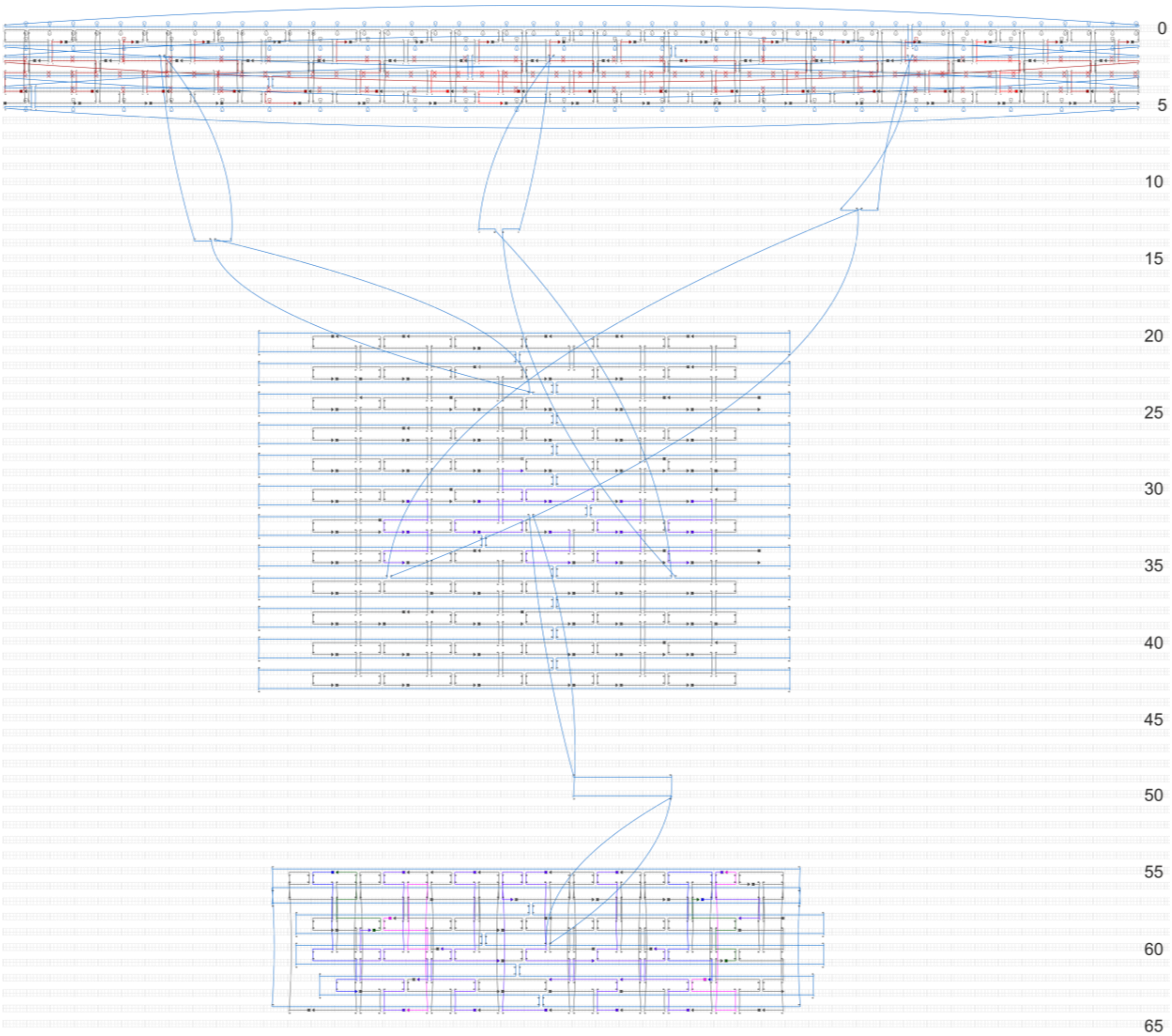

Supplementary Figure 11 | Strand routing diagram of the DNA origami structure for the autonomous plasmonic nanoclock. Staples for two foot strands (green), two upper locking strands (magenta), 16 footholds (positions for lower locking strands and substrates, red) and 20 capture strands (violet). Two blue strands represent the staples revised in the autonomous design.

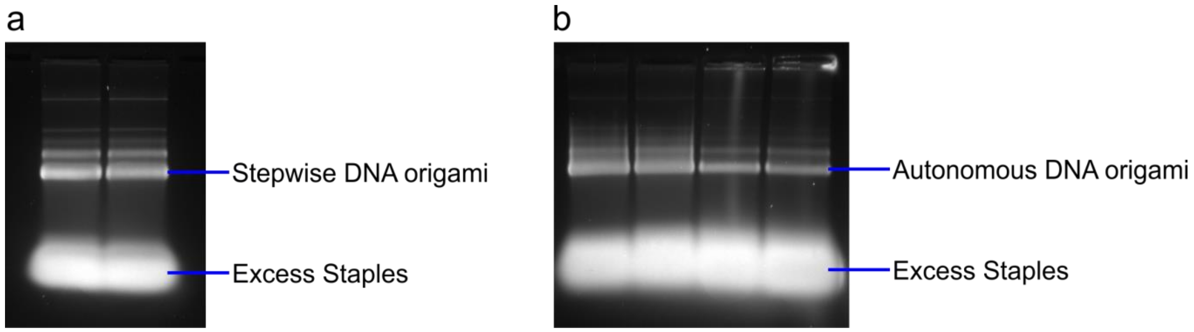

Supplementary Figure 12│Characterization and purification of the DNA origami structures by agarose gel electrophoresis. **a**, DNA origami for assembly of the stepwise plasmonic nanoclocks. **b**, DNA origami for assembly of the autonomous plasmonic nanoclocks.

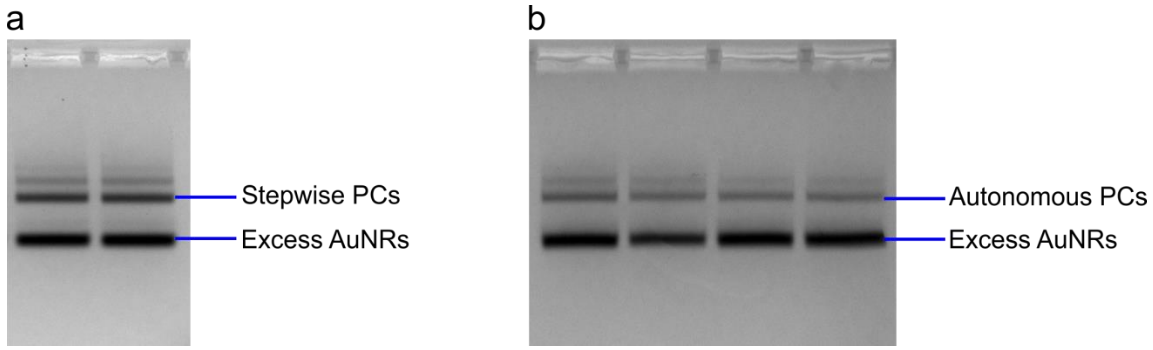

Supplementary Figure 13│Characterization and purification of the plasmonic nanoclocks by agarose gel electrophoresis. **a**, Stepwise plasmonic nanoclocks. **b**, Autonomous plasmonic nanoclocks. PCs: plasmonic nanoclocks

Supplementary Table 1 | Sequences of the stepwise plasmonic nanoclocks.

Unmodified staples	Sequence (5′-3′)
2[135]	CAAGAGAATCGATGAAGTTTA
1[270]	ATTCTTACCAGTATAAAGACC
57[151]	CCAGACGTCGGTGGTGCGGTTTCT
35[99]	AATAAAGGAACCCATGTACCGAACGCCT
41[119]	TCACCCTAGGAAGTTTCCATTACTCATC
39[98]	GTTGAAACCATCGCCCACGCAGGAGTTA
41[203]	CTTGACAGGAACCGAACTGACAAATCCG
43[182]	GATAAATTGTGTCGCAACTTTTCATCAA
37[203]	CACATTCGGGAAGAAAAATCTATCATTG
37[140]	AAAGTTTTCTGTATGGGATTTCCTTTAA
37[98]	GTAGCATGAGTGAGAATAGAATTTTCAC
29[140]	GTAAAACCGCCACGGGAACGGCAGAAAC
27[119]	ATCGCACCTGTTGGGAAGGGCACGCCAG
39[140]	TTGTATCTAAACAGCTTGATAGAAAGAC
27[203]	AAATACAAACCGAGGAAACGCTCTTACC
39[182]	TAATTTCGACGAGAAACACCAAGGCTGG
41[140]	AGCATCGAGGACTAAAGACTTATTATAC
29[182]	AATAGCACACAAGAATTGAGTGAGAATA
35[182]	ATTACCGCTCATCGAGAACAAAGATTCA
26[118]	TGGGATAGGTCACGTTCGCGTAAAATTC
23[98]	AAGAGAAGCATTAAATTTTTGGCCATCA
31[98]	GTGGTGATAAAAGTTTGAGTAAGAAGGA
25[98]	AAAATAATTGGTGTAGATGGGGGACGAC
27[182]	CAGTATGACGGAATACCCAAAACAATGA
25[140]	ACATTAAGTGGGAACAAACGGAGCTTTC
37[182]	TCAGTTGTAAAACGAACTAACGATGGTT
41[98]	AAGGCCGTAAAATACGTAATGGGCAAAA

29[119]	GGTTTTCCACCGGAAACAATCGCTCTCA
25[182]	GACATTCCAATAGAAAATTCATATTACG
41[182]	CTGACCTGAAAGAGGACAGATATCGCCT
60[174]	GTCAATACGATGCTAGAATGCCAACGGC
2[9]	TAGCTTTACCCTGACGAAGCAAACTCCC
27[140]	CGGCACCTTCGCCATTCAGGCCGACGTT
39[203]	TGAATTATCAGTGAATAAGGCGAGTAAT
27[98]	GACAGTAGCGGGCCTCTTCGCGCAAGGC
25[203]	GGGAAGGATAAGTTTATTTTGACGTAGA
23[182]	CCGGAAATTAGAGCCAGCAAAAAAGGGC
20[202]	CCACCAGGGAACCGCCTCCCTGTCAGAC
29[98]	GATTAAGCGTACAGCGCCATGTTTCTCC
33[98]	GCGGAATATAATGGAAGGGTTAAACAGA
2[324]	ACCATAAATCAAAAACATAAAGAAAATTA
2[240]	CACTGCGGAATCGTGGATAAATGGGGCGC
2[93]	AATACAAAGGCTATATTAATGGTAGCTCA
2[261]	CAGTATTCATTGAATAGCCTTGCATCAATT
2[219]	AGAACTGGATAGCGTCTCATAACCTGTTTA
2[156]	GTTGAGAGGCTTTTGAAAGGCTGATTCCCA
2[30]	ATCAGTCAGAAGCAACTTCAATAATTGCTC
2[282]	CTCCAAATGCTTTAATGTAATAAGCATTAA
2[303]	CACCAGAAAACGAGAACCAAATACAGGCAA
2[51]	AATTGCATCAAAAAGATATCGCTCATTTTT
2[114]	AATATTGCCTGAGAGCAACCGTGCAACTAA
2[177]	CCAAAGTTTTGCCAGTAAAGATTAGATTTA
64[223]	CACACGATGATAGCACCACCAGCAGAAGTCA
5[87]	GAATATCCAATCCAATAAACAACATGTTGTC
5[22]	TAGAGAAAATTTAATTTCGAGCCAGTAAGTGT
5[169]	ATTCTGCAATAGTGTTACGAGCATGTAGAATT

63[203]	TTTAATGCGCGAACCCAGTAAGTCAAAGTTGC
5[316]	GGCAAATAAGAATAATCGCCATATTTAACAGA
5[127]	AGTACGCCTCCGGCACAATAGATAAGTCCTAA
58[230]	ACGTGCCGGCGCTTTCCGCCGGGCGCGGGGCG
5[232]	GCTATACGCTATTAGAATTACCTTTTTTAATTT
5[43]	CTTTTGTTAGTTAATATAAAGTACCGACACCAA
1[144]	CAAGAGACTACCTTTTTAAGTGTCTGGTCAATAT
5[211]	CAAATGAATCCTTGAATTACATTTAACAAAAGA
1[60]	GTATTTTTCAAATATATTATAAGAGGGTTTTAA
5[148]	TTCCATAAGGTCTGAGAAAAATAATATCCCCCTC
5[190]	GTTTGACTTAGATTACAATAATCGGGAAAACAAA
5[295]	CATCCAATTACTAGATCAACAGTAGGGCTTCCGC
5[64]	GCGGATGGAGAAAACAAGTAATTCTGTCCAGGTT
5[106]	ACATGTTAACTATATATGCAGAACGCGCCTCGGT
1[186]	AATAGACGCTGAGAAGAGTCGAACGAGTCAAAAG
1[249]	AAATGCTTCTGTAAATCGTTTTTCATTAATTTTT
5[253]	GAGCTGAAATAACCTCAGTACATAAATCAACGCG
1[39]	GAATTTCATCTTCTGACCTGTACCTTAGCGAACC
1[102]	CTAGTAAATGCTGATGCAAATAATGCTCCGGAGA
1[228]	TTTATTAATTTTCCCTTAGGTCAATATATTTTAA
1[333]	CCATAAATAAGGCGTTAAAGAATTAGCCTAAATC
1[81]	CGAGCAAGACAAAGAACGCGCTTAGACGAAAGAC
1[291]	CGCAAAAGCCTGTTTAGTAATAGTAGTCTTTTGC
1[123]	TCATTAGGTTGGGTTATATTTAAATATTCTAGCT
1[207]	ATTAAAACATAGCGATAGCCATTAGAAGTAATGT
22[202]	TGTAGCGAATGAAACCATCGACCGACTTGAGCCAT
1[165]	TAATAATTTATCAAAATCATTAACAGTCGGAGACA
61[147]	CGTTGGCCGCACAGGCGGCCAGCCCGAGGATTGCC
23[105]	GGATTAGCTGCCTATTTTGATCCTCATTAAAGCCA

34[195]	TGCGGGAGCCTAATAAATGAAAACAGGGAAGCGCA
1[312]	GAGAAACACCGGAATCATAATAAATCAAACATTAT
35[203]	CAAATCAAGAGCAACACTAAGTACCGCACGCCCAA
59[98]	AGTTGAGAATTCCATGGTTCCGGTCCACGCTGGTT
59[217]	GCTGGCAGCCTCCGTGAGGAAAACCGTCCACCAGT
43[203]	CGACCTGCTCCATGTCATAAGAGAACCGTGCTCAT
29[203]	GAAGCCCGCTAATATCAGAGAGATAACCATAGCTA
59[147]	GTGATGAGCGGCGGGCCGTTTTGGTGCTCATCCCT
40[195]	TTGCCCTAACTTTAACGTTAAAGATTTAGGAATAC
39[112]	AAAAAAAGGCTCCATTGCGCCGACAATGGGGATCG
5[0]	AGCAATAACCGTGTGAACATGTAATTTAGGCGGTT
2[202]	CAACTTAATAGTAAAATATGCCTGTACATTTCG
28[195]	AATAATATTAGCAATCACAATAACCGATTGAGGGA
57[126]	GCCTGTGCACTCTGTCACGGTAATCATGGTCTTTA
1[18]	GCATTGGTTTGAAATACCGAAGCAACAGGTCAGGAT
62[121]	CTGGTATTGGGCGCCAGGCCTGGCAAATCAAGTGTGTT
55[95]	TGGCACAACAAAGCATAAAGTGTATTAATGAGCGCGGG
58[114]	CAGGGGTACCGAGCTCGGTGAAATCCCTTATCCTGAGA
55[221]	AAAGGTTATCATATCTGGTCAGTTCGAACGACCTAAAA
60[128]	TGTAATTCGTCATACCGGGGGTTTACTGCGCCAGCAAA
60[191]	ACTCAAACGCGTCTGGTCAGCAGCAGCTTACCTGCGGC
2[72]	GATAGGAAGCTTTGAGAGATCTCATATGTACCCCGACGA
63[147]	TCACCAGTGAGATAACGCTGAGACGCTCACTGCCCTTTCTTT
34[139]	CAATATACAGATGAATATACAAACGGATTCGCCTGACGATCT
29[210]	TTTTTAAAGAAGGATACATAACCACGGATAAATATTGACGGA
20[181]	CCGCCGCACCGGAACCAGAGCATCGGCATTTTCGGAGCAAGG
24[132]	TGTAAACTGCTCAGCGTATAACTGGTAAGATATTCACAAACA
38[153]	TGAATTTTGTCGTCAAACAATGTAACAGTTGCACCCAGCTAT
21[140]	TAACGGGGTCAGGCGCAGGTCAGACGATTGGCCTTTAAGTTT

63[161]	AGAATACGTGGCACTTCTGACGTTTGGAGGGTAAACATCCCA
58[160]	AGCACATAGGGTAAAGTTAAAGATAATATGTTCCACTGAAAG
30[132]	ATAACCTCCAGTCATGCGCAATCCAGCCCGGATTGGCCAGCT
20[160]	GGTTGAGCATCTTTTCATAATCCCCTTATTAGCGTCGAGAGG
20[118]	AATAAATGATACAGGAGTGTAACAGTTAATGCCCCGATTAGC
43[140]	CAAGCGCGAAACAAGGCTTTGGAACGAGAATTTCTGGTTTAT
41[168]	GGCGCATGAACGAGTAGTATTCGAGGTGGGTAGCAACGACCA
39[168]	GGCTTGAGGAACAACATTAGTTAGTAAACAGCTTGCTAATTG
63[182]	ATTTTTGAATGGCTTGGCCAATTAAAGATGTTGCCGGCTGGA
22[139]	CAGTGCCTACCAGGCGGATAAGCAAATATTTAAATTTCATCA
43[98]	GAATACACTAAAACAAACGGGCTTTTGCACAACAAATCTCCA
20[97]	GAATGGAGCGTCATACATGGCTTTCGGAACCTATTACTCCTC
23[161]	GTACCATTACCATTTCATAGCCAAAATCCAGCATTGACAGGA
39[210]	CCTTATGGGACGTTAACTAATCATAGTAGATATAGAAGGCTT
37[168]	GGTAGAAGCAAGCCGTTTTCATCGGGAGTTTCCAGACTTACA
43[168]	TTGTATCGAACGGTGTACAGGCTACAGAAGTACAACGGAGAT
25[161]	CAGCGCCAAAGACAATCACCAGTAGCTGATATAATCGAGTAA
24[195]	TTGGGAACGTCACCCGTTTTCCACCACCAACCACCACCAGAG
34[223]	GCGAGGCGTTTTAGATAAACACGATTTTGGAGAATATTGAGC
27[161]	AAATTAAGACTCCTTATGGTTTACCAACCCGTCGGGTGCCGG
23[119]	GGGGTTTGTTAATATTTTGTTCTGGCCTTCCTGTAACCGTAA
35[119]	TTCAGGTTGAATACCAAGTGCACAGCCCTCATAGTCAACTTT
29[161]	AGTAAGAGCAAGAAAGAACTGGCATGACCAGGCAAAGTGCCA
5[274]	CTACTATCATATGCGTTATATATGTGAGTGAAAGGTGTATTTCAA
63[105]	GAGAGGCAGCAAGCGAAATCGCGGCATCTCCAGCGCAGTGTCCTGC
32[111]	TTAATTTAGGGATAGGCGAAATTGGGTAGATCGGTTCGGCCTCAGGAAG
38[118]	CAACAGTTTCAGCGTCCACAGCCAATAGAAATTGCCTTCTGATATCATC
57[196]	GGTGTCCGGTGCTGGGTCCGTTTTTTCGTCTTTAGCTCCAACTAAAAGG
57[175]	CGCAACCAACCGCAGATTGCCGTTCCGGAATAGATTCCACTACAGAGAT

24[223]	AATTATCACCGTCATAGCAGCCTTTAGCCAGAGCCCACCCTCAGAGCCG
37[126]	TAGCGTAATTGCTTTTAACGTATCCTGACAGATGAAAATCCTAGAGACG
64[90]	TGCCCCAATCCTGTCTGCAGCGGTGCCCCCTGCATCTTCGCGTCCTCAC
43[119]	TTTGACCCCCAGCGTTTCATGCAGCAGCCCGATAGAAAGGAGTGCTAAA
35[168]	TCATCGTAAATCAAGCTAACGCAGCCAGCAGTTGGGCGGTCATGAATCT
28[153]	AGCGCCAGCTTCTGATTCTCCATGTGAGTGTATAAGTGCCGTTTTGCCTTGAGTAA
Capture strands	Sequence (5′-3′)
31[119]	AAAAAAAAA CGGAAAATTGCCCGAACGTTAATATTCC
31[140]	AAAAAAAAA AGCGGATCGACAACTCGTATTTGGCAAT
31[161]	AAAAAAAAA TGCTCATTTGCCGATAAGCCCAATAACTTTCAGAGAAATTTC
31[182]	AAAAAAAAA ACATAAAAATAGCAGCCTTTAAGCGTCT
31[203]	AAAAAAAAA TTAGACGTTGTTTAACGTCAATTGCCAG
33[119]	AAAAAAAAA TGATTATTTGTTTGGATTATAGTAGATT
33[140]	AAAAAAAAA TCACAATTTTATCCAACAATTCAAACTTGTGGAGCGACGGCC
33[161]	AAAAAAAAA TACCAACGATTAGTTGCTATTTACCTTTTATATTT
33[182]	AAAAAAAAA TTCCAGAGGTTTTGAAGCCTTAGGAATC
33[203]	AAAAAAAAA TTACAAACGAACCTCCCGACTTAGCAAG
56[118]	AAAAAAAAA TCGTTAAGCAAAATTGTTATCGGGTGCCTAATGAGAAAC
56[139]	AAAAAAAAA TACACTGAAGAATATAGCTGTACTCACATTAATTG
56[160]	AAAAAAAAA TTGCTCGTGAGTGTAAAAAGCCAGCAGCAAATGAACAGTGCC
56[181]	AAAAAAAAA TGGTAATACAAGAGTAGAGCCAAGCATCACCTTGCCAGTATT
56[202]	AAAAAAAAA GGGTCACACGTGGAGAGCACTCAAATATCAAACCCATAAAAC
64[118]	AAAAAAAAA GAGTTGCGGTTTGCTCGTGCCAGCTGCAAAGCCTGCGCTCACGATC
64[139]	AAAAAAAAA CTTCACCGGTGGTTGCTTTCCAGTCGGGTGAGCTATTCC
64[160]	AAAAAAAAA CGCGGGCAACAGCTATAGGGTTCATAAAGCGG
64[181]	AAAAAAAAA AGAACCCAGACAATAACACCGCCTGCAAAAATCTA
64[202]	AAAAAAAAA GACATTCATTAGTCAGAGGTGAGGCGGTTGAACCTAACA
Footholds	Sequence (5′-3′)
4[5] $fh_{3'}$	TCTGAATGCTGGATCTC TCAGAGTCAGGTTACCGCCACCCTCAACG

4[26] $fh_{4'}$	TCTGAATGCGACTCTAA AGACCGTATTATACCGTACTCAGGAAGAG
4[47] $fh_{5'}$	TCTGAATGCGACGAGTT TTCGAGAGCGGAACGCCCGGAATAGTAAGA
4[68] $fh_{6'}$	TCTGAATGCGGTCTAAT TTCAAATTAAGAATCAGAAAAGCCAAAG
4[89] $fh_{7'}$	TCTGAATGCTAGAAGTC GGGTAGCTATTCGCTTAATTGCT
4[110] $fh_{8'}$	TCTGAATGCAATAGTCC GATAACAGGTCCGTAAAACTAGCATCAG
4[131] fh_1	TCTGAATGCGAACTGGT GATATTTCTGGAGCAAAAAACCAAATCACCAAAGTTTCA
4[152] fh_2	TCTGAATGCACGCAAGG GTCAAAATAGCTACCAGACGACGATGAA
4[173] fh_3	TCTGAATGCTGGATCTC GGTGAGCAAAAGAGAACGGGTATTCATCC
4[194] fh_4	TCTGAATGCGACTCTAA GTAGGAGGGGGGTCTTTCCTTATCAACC
4[215] fh_5	TCTGAATGCCACGAGTT ATGCAGTTTAGTGATGAAACAAACAT
4[236] fh_6	TCTGAATGCGGTCTAAT AGAACCCCAATAATTACCTGAGCAATTTCA
4[257] fh_7	TCTGAATGCTAGAAGTC CGCAACATAAAAGGCGAATTATTCATGG
4[278] fh_8	TCTGAATGCAATAGTCC GGGAGACCCCCTATTTTCAGGGAATTACAA
4[299] $fh_{1'}$	TCTGAATGCGAACTGGT GACCCACAGTTCCTCAGAGCCACCCCAA
4[320] $fh_{2'}$	TCTGAATGCACGCAAGG GGTTGTATGACCGCCACCCTCAGAAAATT
Feet	Sequence (5′-3′)
59[109]F1N	GCATTCAGATTT CCCCACGCGTGCCTGTTCAGACGAAGATGCCGGGTTACTTGA
61[213]F2N	GCATTCAGATTT ATCATAAAATATCTCGTCTAGAACGTCAGCGTAGCATCAATGAGCC
Blocking strands	Sequence (5′-3′)
B_1	ACCAGTTCGCATTCTCTAGCTTACT
B_2	CCTTGCGTGCATTCTCTCCTTATCG
B_3	GAGATCCAGCATTCTTTGTACGAAC
B_4	TTAGAGTCGCATTCTATTAGCAACG
B_5	AACTCGTCGCATTCTCTCACTAATT
B_6	ATTAGACCGCATTCTTTGAGTTCCG
B_7	GACTTCTAGCATTCCTTCACTTTCA
B_8	GGACTATTGCATTCGTTGTAGATCC
Removal strands	Sequence (5′-3′)
R_1	AGTAAGCTAGAGAATGCGAACTGGT

R_2	CGATAAGGAGAGAATGCACGCAAGG
R_3	GTTCGTACAAAGAATGCTGGATCTC
R_4	CGTTGCTAATAGAATGCGACTCTAA
R_5	AATTAGTGAGAGAATGCGACGAGTT
R_6	CGGAACTCAAAGAATGCGGTCTAAT
R_7	TGAAAGTGAAGGAATGCTAGAAGTC
R_8	GGATCTACAACGAATGCAATAGTCC

Supplementary Table 2 | Additions of blocking and removal strands for a full-turn clockwise rotation. All the fuel strands have a concentration of 250μM. So do those in Supplementary Table 3 and 4.

Steps	Strands added	Steps	Strands added
1 → 2	0.4 μL B_1 and 0.4 μL R_2	1′ → 2′	1.2 μL B_1 and 0.8 μL R_2
2 → 3	0.8 μL B_2 and 0.4 μL R_3	2′ → 3′	1.2 μL B_2 and 0.8 μL R_3
3 → 4	0.8 μL B_3 and 0.4 μL R_4	3′ → 4′	1.2 μL B_3 and 0.8 μL R_4
4 → 5	0.8 μL B_4 and 0.4 μL R_5	4′ → 5′	1.2 μL B_4 and 0.8 μL R_5
5 → 6	0.8 μL B_5 and 0.4 μL R_6	5′ → 6′	1.2 μL B_5 and 0.8 μL R_6
6 → 7	0.8 μL B_6 and 0.4 μL R_7	6′ → 7′	1.2 μL B_6 and 0.8 μL R_7
7 → 8	0.8 μL B_7 and 0.4 μL R_8	7′ → 8′	1.2 μL B_7 and 0.8 μL R_8
8 → 1′	0.8 μL B_8 and 0.8 μL R_1	8′ → 1	1.2 μL B_8 and 1.2 μL R_1

Supplementary Table 3 | Additions of blocking and removal strands for the plasmonic nanoclock from position 5-5′ to position 5′-5 during the time-course CD measurements.

Steps	Strands added
5 → 6	0.4 μL B_5 and 0.4 μL R_6
6 → 7	0.8 μL B_6 and 0.4 μL R_7
7 → 8	0.8 μL B_7 and 0.4 μL R_8
8 → 1′	0.8 μL B_8 and 0.4 μL R_1
1′ → 2′	0.8 μL B_1 and 0.4 μL R_2
2′ → 3′	0.8 μL B_2 and 0.4 μL R_3
3′ → 4′	0.8 μL B_3 and 0.4 μL R_4
4′ → 5′	0.8 μL B_4 and 0.8 μL R_5

Supplementary Table 4 | Additions of blocking and removal strands for a 15-step counterclockwise rotation followed by a 9-step clockwise rotation.

Counterclockwise Steps	Strands added	Clockwise Steps	Strands added
1 → 8′	0.4 µL B_1 and 0.4 µL R_8	2 → 3	1.2 µL B_2 and 1.6 µL R_3
8′ → 7′	0.8 µL B_8 and 0.4 µL R_7	3 → 4	2 µL B_3 and 1.6 µL R_4
7′ → 6′	0.8 µL B_7 and 0.4 µL R_6	4 → 5	2 µL B_4 and 1.6 µL R_5
6′ → 5′	0.8 µL B_6 and 0.4 µL R_5	5 → 6	2 µL B_5 and 1.6 µL R_6
5′ → 4′	0.8 µL B_5 and 0.4 µL R_4	6 → 7	2 µL B_6 and 1.6 µL R_7
4′ → 3′	0.8 µL B_4 and 0.4 µL R_3	7 → 8	2 µL B_7 and 1.6 µL R_8
3′ → 2′	0.8 µL B_3 and 0.4 µL R_2	8 → 1′	2 µL B_8 and 1.6 µL R_1
2′ → 1′	0.8 µL B_2 and 0.8 µL R_1	1′ → 2′	2 µL B_1 and 1.6 µL R_2
1′ → 8	1.2 µL B_1 and 0.8 µL R_8	2′ → 3′	2 µL B_2 and 2.4 µL R_3
8 → 7	1.2 µL B_8 and 0.8 µL R_7		
7 → 6	1.2 µL B_7 and 0.8 µL R_6		
6 → 5	1.2 µL B_6 and 0.8 µL R_5		
5 → 4	1.2 µL B_5 and 0.8 µL R_4		
4 → 3	1.2 µL B_4 and 0.8 µL R_3		
3 → 2	1.2 µL B_3 and 0.8 µL R_2		

Supplementary Table 5 | Track arrangements of the autonomous plasmonic nanoclocks for clockwise rotation.

	Locking strands	Substrates
Position 1–2	fh_1 and $fh_{1'}$	@ fh_2 and $fh_{2'}$
Position 1–2–3	fh_1 and $fh_{1'}$	@ fh_2–fh_3 and $fh_{2'}$–$fh_{3'}$
Position 1–2–3–4	fh_1 and $fh_{1'}$	@ fh_2–fh_4 and $fh_{2'}$–$fh_{4'}$
Position 1–2–3–4–5	fh_1 and $fh_{1'}$	@ fh_2–fh_5 and $fh_{2'}$–$fh_{5'}$
Position 1–2–3–4–5–6	fh_1 and $fh_{1'}$	@ fh_2–fh_6 and $fh_{2'}$–$fh_{6'}$
Position 1–2–3–4–5–6–7	fh_1 and $fh_{1'}$	@ fh_2–fh_7 and $fh_{2'}$–$fh_{7'}$

Supplementary Table 6 | Track arrangements of the autonomous plasmonic nanoclocks with open sites.

	Locking strands	Substrates
Position 1–2–3–x–5	fh_1 and $fh_{1'}$	@ fh_2–fh_3, fh_5 and $fh_{2'}$–$fh_{3'}$, $fh_{5'}$
Position 1–2–x–x–5	fh_1 and $fh_{1'}$	@ fh_2, fh_5 and $fh_{2'}$, $fh_{5'}$

Supplementary Table 7 | Track arrangements of the autonomous plasmonic nanoclocks for counterclockwise rotation.

	Locking strands	Substrates
Position 1–8′	fh_1 and $fh_{1'}$	@ fh_8 and $fh_{8'}$
Position 1–8′–7′	fh_1 and $fh_{1'}$	@ fh_8–fh_7 and $fh_{8'}$–$fh_{7'}$
Position 1–8′–7′–6′	fh_1 and $fh_{1'}$	@ fh_8–fh_6 and $fh_{8'}$–$fh_{6'}$
Position 1–8′–7′–6′–5′	fh_1 and $fh_{1'}$	@ fh_8–fh_5 and $fh_{8'}$–$fh_{5'}$
Position 1–8′–7′–6′–5′–4′	fh_1 and $fh_{1'}$	@ fh_8–fh_4 and $fh_{8'}$–$fh_{4'}$
Position 1–8′–7′–6′–5′–4′–3′	fh_1 and $fh_{1'}$	@ fh_8–fh_3 and $fh_{8'}$–$fh_{3'}$

Supplementary Table 8 | Sequences of the functional strands for autonomous rotation.

Substrate_footholds	Sequence (5′-3′)
4[5] $Sub_{3'}$	GAGTTGG rA TAGGTTATGAGATTGTT TCAGAGTCAGGTTACCGCCACCCTCAACG
4[26] $Sub_{4'}$	GAGTTGG rA TAGGTTATGAGATTGTT AGACCGTATTATACCGTACTCAGGAAGAG
4[47] $Sub_{5'}$	GAGTTGG rA TAGGTTATGAGATTGTT TTCGAGAGCGGAACGCCCGGAATAGTAAGA
4[68] $Sub_{6'}$	GAGTTGG rA TAGGTTATGAGATTGTT TTCAAATTAAGAATCAGAAAAGCCAAAG
4[89] $Sub_{7'}$	GAGTTGG rA TAGGTTATGAGATTGTT GGGTAGCTATTCGCTTAATTGCT
4[110] $Sub_{8'}$	GAGTTGG rA TAGGTTATGAGATTGTT GATAACAGGTCCGTAAAACTAGCATCAG
4[152] Sub_2	GAGTTGG rA TAGGTTATGAGATTGTT GTCAAAATAGCTACCAGACGACGATGAA
4[173] Sub_3	GAGTTGG rA TAGGTTATGAGATTGTT GGTGAGCAAAAGAGAACGGGTATTCATCC
4[194] Sub_4	GAGTTGG rA TAGGTTATGAGATTGTT GTAGGAGGGGGGTCTTTCCTTATCAACC
4[215] Sub_5	GAGTTGG rA TAGGTTATGAGATTGTT ATGCAGTTTAGTGATGAAACAAACAT
4[236] Sub_6	GAGTTGG rA TAGGTTATGAGATTGTT AGAACCCCAATAATTACCTGAGCAATTTCA
4[257] Sub_7	GAGTTGG rA TAGGTTATGAGATTGTT CGCAACATAAAAGGCGAATTATTCATGG
4[278] Sub_8	GAGTTGG rA TAGGTTATGAGATTGTT GGGAGACCCCCTATTTTCAGGGAATTACAA
4[320] $Sub_{2'}$	GAGTTGG rA TAGGTTATGAGATTGTT GGTTGTATGACCGCCACCCTCAGAAAATT
DNAzyme_feet	Sequence (5′-3′)
59[109]	CATAACCTA CCGAGCCGGTCGAATCAACTC TCTT CCCCACGCGTGCCTGTTCAGACGAAGATGCC
61[213]	CATAACCTA CCGAGCCGGTCGAATCAACTC TCTT ATCATAAAATATCTCGTCTAGAACGTCAGCGTAGC
58[114]locking2	GCATTCAGATTT CAGGGGTACCGAGCTCGGTGAAATCCCTTATCCTGAGA
62[206]locking1	GCATTCAGATTT AAACTTTAATGCGCGAACCCAGTAAGTCAAAGTTGC
Removal strand r	GTATCTGTCGT ACCTACCGAGCCGGTCGAA
Blocking strand b	TTGATTCGACCGGCTCGGTAGGT ACGACAGATAC
Revised staples	Sequence (5′-3′)
56[97]	GGGTTACTTGATGGCACAACAAAGCATAAAGTGTATTAATGAGCGCGGG
57[206]	ATCAATGAGCCGGGTCACACGTGGAGAGCACTCAAATATCAAACCCATA
56[212]	AAAAAAAAA GGTGCGGGGTCATTGCAGGACTTG

Supplementary Table 9 | Additions of the trigger strands to actuate the autonomous plasmonic nanoclocks.

Steps	Strands added
1	100 µM 1 µL removal strand r
2	250 µM 0.5 µL B_1
